# Bridging the gap between pragmatic intervention design and theory: using behavioural science tools to modify an existing quality improvement programme to implement “Sepsis Six”

**DOI:** 10.1186/s13012-016-0376-8

**Published:** 2016-02-03

**Authors:** Siri H. Steinmo, Susan Michie, Christopher Fuller, Sarah Stanley, Caitriona Stapleton, Sheldon P. Stone

**Affiliations:** 1 Department of Clinical, Educational and Health Psychology, University College London, London, UK; 2 Department of Infectious Disease Informatics, Farr Institute, University College London, London, UK; 3 Royal Free Hospital, Pond Street, London, UK; 4 University College London Medical School, Rowland Hill Street, London, UK

**Keywords:** Implementation intervention, Quality improvement, P-D-S-A cycle, Theoretical Domains Framework, Behaviour Change Technique Taxonomy, Sepsis Six, Sepsis, Health professional behaviour

## Abstract

**Background:**

Sepsis has a mortality rate of 40 %, which can be halved if the evidence-based “Sepsis Six” care bundle is implemented within 1 h. UK audit shows low implementation rates. Interventions to improve this have had minimal effects. Quality improvement programmes could be further developed by using theoretical frameworks (Theoretical Domains Framework (TDF)) to modify existing interventions by identifying influences on clinical behaviour and selecting appropriate content. The aim of this study was to illustrate using this process to modify an intervention designed using plan-do-study-act (P-D-S-A) cycles that had achieved partial success in improving Sepsis Six implementation in one hospital.

**Methods:**

Factors influencing implementation were investigated using the TDF to analyse interviews with 34 health professionals. The nursing team who developed and facilitated the intervention used the data to select modifications using the Behaviour Change Technique (BCT) Taxonomy (v1) and the APEASE criteria: affordability, practicability, effectiveness, acceptability, safety and equity.

**Results:**

Five themes were identified as influencing implementation and guided intervention modification. These were:(1) “knowing what to do and why” (TDF domains *knowledge*, *social/professional role and identity*); (2) “risks and benefits” (*beliefs about consequences*), e.g. fear of harming patients through fluid overload acting as a barrier to implementation versus belief in the bundle’s effectiveness acting as a lever to implementation; (3) “working together” (*social influences*, *social/professional role and identity*), e.g. team collaboration acting as a lever versus doctor/nurse conflict acting as a barrier; (4) “empowerment and support” (*beliefs about capabilities*, *social/professional role and identity*, *behavioural regulation*, *social influences*), e.g. involving staff in intervention development acting as a lever versus lack of confidence to challenge colleagues’ decisions not to implement acting as a barrier; (5) “staffing levels” (*environmental context and resources*), e.g. shortages of doctors at night preventing implementation.

The modified intervention included six new BCTs and consisted of two additional components (Sepsis Six training for the Hospital at Night Co-ordinator; a partnership agreement endorsing engagement of all clinical staff and permitting collegial challenge) and modifications to two existing components (staff education sessions; documents and materials).

**Conclusions:**

This work demonstrates the feasibility of the TDF and BCT Taxonomy (v1) for developing an existing quality improvement intervention. The tools are compatible with the pragmatic P-D-S-A cycle approach generally used in quality improvement work.

**Electronic supplementary material:**

The online version of this article (doi:10.1186/s13012-016-0376-8) contains supplementary material, which is available to authorized users.

## Background

Reducing mortality from sepsis, a major cause of death from infection through shock and multiple organ failure, is a national and international priority [1–3]. Mortality can be halved if sepsis is treated within an hour of presentation with the evidence-based guidelines, “Six Steps of Sepsis Treatment”, known as the “Sepsis Six” clinical care bundle. The steps are measuring lactate levels and urine output, administering antibiotics, oxygen and fast intravenous fluids (a fluid challenge) and taking blood cultures. Despite this, published results of a UK audit suggest that implementation is low [4] and interventions to improve this have shown small and un-sustained effects, which are often difficult to replicate [5, 6].

There is widespread recognition that theory should be used to inform the development of interventions that aim to encourage implementation of evidence-based guidelines by health professionals [7]. In practice, however, clinical practitioners often find theory “abstract, intimidating and irrelevant” [8, 9], so most quality improvement programmes are developed pragmatically. Lack of effectiveness of such interventions may be due in part to the lack of explicit behavioural theory at the design stage [10–12].

A second problem facing interventions to improve implementation is the poor specification and reporting of their content. Descriptions of interventions are rarely comprehensive, and there is a lack of common definitions to describe them [12–15]. The lack of theoretical rationale and reporting of content may make it difficult to derive maximum patient benefit because (a) potential determinants of health professional behaviour change may not have been identified and targeted and (b) scope for replication or improvement is limited if what has been delivered has not been fully described.

Problems of implementation, lack of explicit theory in intervention development and partial intervention description were evident in relation to an intervention to improve implementation of the Sepsis Six in a UK National Health Service (NHS) hospital in London [16]. The intervention was originally introduced by a specialist Patient at Risk and Resuscitation nursing team and developed collaboratively and iteratively by front-line staff and one of the team, a Patient Safety Facilitator (NHS nurse Band 7). The pragmatic Plan, Do, Study, Act (P-D-S-A) cycle approach [17] was used to introduce the intervention to pilot areas in small, incremental steps and respond to staff feedback about challenges facing implementation and the intervention itself. The P-D-S-A cycle, derived from improvement science methods within manufacturing and economics [18], implements an intervention in one setting, evaluates it, and adjusts it by discarding/adding content according to local context and with input from those receiving the intervention before repeating the process elsewhere. P-D-S-A is widely used in quality improvement interventions, but lacks a theoretical basis and there is little evaluation of the content and application of such programmes [19, 20].

The Sepsis Six implementation intervention has now reached six clinical areas and whilst it has achieved considerable success, with implementation of the bundle rising from 20 to 80–90 % and mortality from sepsis dropping from 22 to 12 % at the time of data collection (unpublished data c/o Royal Free Hospital Patient at Risk and Resuscitation Team, Devaney, Stapleton, Stanley, 2013). Improvements took 4 years and involved numerous P-D-S-A cycles (e.g., 45 iterations of the bundle protocol document in Accident & Emergency alone). The hospital’s target of implementation of the bundle for at least 95 % of patients with a source of infection and two physiological symptoms (“triggers for sepsis”) is still not consistently reached and sustained. This raises the question of how best to develop the implementation intervention before extending it elsewhere.

The intervention consisted of introductory group education and training, target-setting, implementation and patient outcome data measurement, group and individual staff feedback and promotional documents and materials to aid implementation such as a “sepsis trolley” or “sepsis bag” containing the necessary equipment. Its content and potential mechanisms of action have been described elsewhere [16] using tools from behavioural science to make theory more accessible to clinical practitioners: the Theoretical Domains Framework (TDF) [21] and the Behaviour Change Technique Taxonomy [22]. The TDF is a framework that brings together key theoretical constructs derived from a wide range of behaviour change theories. It identifies 14 key domains, for example *knowledge* and *social influences*, which shape health professional behaviour and are possible targets for intervention. The Behaviour Change Technique Taxonomy (v1) is a structured list of 93 techniques to change behaviour. Behaviour change techniques (BCTs) are seen as the “active ingredients” of behaviour change interventions, defined as irreducible, replicable components that have potential to directly impact behaviour [23].

The TDF has been used to study a range of health professional behaviours [24–29] and together with the BCT Taxonomy (v1) can be used for understanding influences on a given behaviour and selecting appropriate strategies for intervention [23]. The BCT Taxonomy (v1) has also been used to identify active ingredients in other existing implementation interventions. Both tools have been used to inform the design of various implementation interventions in healthcare settings [30, 31]. In the current study, an existing pragmatically designed quality improvement programme, which had achieved some level of success, was already ongoing. It is not clear whether the TDF and BCT Taxonomy (v1) can be used as tools to classify perceived barriers and facilitators and thus aid the modification process of existing interventions such as this. This is important because existing quality improvement programmes are often in need of enhancement, rather than design from the outset.

The study aims to test the usefulness of these behavioural science tools for bridging the gap between theory and pragmatic clinical practice by (a) investigating influences on implementation of the Sepsis Six clinical care bundle and (b) modifying and reporting intervention content.

## Methods

### Step 1: qualitative interview study: identifying influences on Sepsis Six implementation

#### Participants

Participants were a convenient sample of nurses, doctors and midwives at various levels of seniority working in six clinical areas. All participants had at least one experience of implementing the Sepsis Six. The study protocol was submitted for ethical review to the Hampshire B research ethics committee. Since the study was considered to be service evaluation or audit, and involved participants who were being recruited by virtue of their professional role, it did not fall in to the Governance Arrangements for Research Ethics Committee’s requirements for review. All participants gave written consent for interviews to be recorded and transcribed.

#### TDF interviews

A semi-structured/focus group interview topic guide (see Additional file 1) was developed to explore the relevance of 14 TDF domains to the implementation of the bundle. Questions were informed by examples from the original TDF paper [10] and suggestions from the Patient at Risk and Resuscitation nursing team who introduced the intervention. The topic guide was developed iteratively via pilot interviews.

Seventeen individual interviews and three focus groups (ranging from 2 to 10 participants) were carried out. This was done partly for pragmatic reasons (to increase the participation rate in busy clinical areas of this working hospital) and partly to benefit from the advantages that both methods offer. Focus groups are useful for generating new thinking about a topic and often lead to rich in-depth discussion. Individual interviews, on the other hand, may lead to responses that are free from the influence of the peer group [32]. Interviewees were asked to describe (1) which activities to promote Sepsis Six had been useful and (2) specific incidences when they cared for a septic patient and the full bundle was not implemented within the 1-h timeframe. Specifically, participants were asked to describe what happened including what “got in the way” and what “would have helped” implementation. The second question was followed by specific prompts relating to influences on implementation, covering all 14 domains of the TDF. Each session, lasting between 20 and 35 min, was audio-recorded and transcribed.

#### Coding and thematic analysis of interview transcripts

Coding guidelines were developed iteratively using the first four transcripts, which were coded in parallel and discussed by researchers CF and SHS. Influences on implementation were assigned to 1 or more of the 14 TDF domains. Where an agreement was not reached, a third researcher with expertise in using the TDF (LA) was consulted. (See Additional file 1 for TDF Coding Guidelines.)

To assess reliability, every fifth transcript was coded by both coders. Cohen’s kappa was calculated. If this did not reach a minimum of *k* = 0.75 (*substantial agreement*) [33], the batch of five transcripts was double-coded and any disagreements were discussed and resolved. Coding guidelines were modified accordingly.

Building on the notes and ideas generated throughout this coding process, SS and CF separately conducted thematic analysis to identify themes within and across domains. Results were compared, and the data were presented and discussed with the Patient at Risk and Resuscitation team until a final consensus was reached.

### Step 2: development of a modified intervention to improve Sepsis Six implementation

#### Generating ideas for intervention modification

We conducted a round-table discussion with the Patient Safety Facilitator, the Patient at Risk and Resuscitation team (*n* = 3), the two study researchers (CF and SHS) and another behavioural scientist with expert experience with the TDF and BCT Taxonomy who was not involved with the study (KS). In order to generate discussion about potential modifications, participants were presented with (a) the frequent domains and themes from the step 1 analysis, (b) a full description of the existing intervention’s content and the potential TDF domains that it targeted [16] and (c) a list of new BCTs suggested by the results of Step 1 [34, 35].

#### Delphi exercise: selection of intervention modifications

Over a two-round Delphi exercise, the four nurses independently rated and commented on whether each modification suggested in step 2 was *affordable*, *practical*, *effective*, *acceptable*, *safe* and *equitable* (the APEASE criteria) [23]. Each nurse assigned a mark of 0 or 1 (no/yes) to each of the six criteria, hence a maximum APEASE score of 24 for each potential modification. There was no cut-off score above which all potential modifications were accepted and below which all were rejected. Instead, the ratings were used to generate discussion and guide decision-making until a consensus agreement for a modified intervention was reached and an intervention protocol was agreed.

Table 1 illustrates the research process of the study and includes steps taken in the previous paper that characterised the intervention before modification [16].

**Table 1:** Study process

Step	Tasks	Behavioural science tools used
Characterise existing intervention (fully described in [16])	Collection of data on intervention content from relevant intervention documents, interviews with intervention facilitators/developers, observation of intervention delivery	Template for Intervention Description and Replication (TIDieR)
	Data analysed for BCTs and intervention functions	BCT Taxonomy (v1), behaviour change wheel
	BCTs and functions mapped to TDF domains likely to be targeted	TDF
Identification of influences on Sepsis Six implementation	Design and piloting of interview topic guide	TDF
	Conduct interviews, analyse data/code responses	TDF, thematic analysis
	Ideas for modification: mapping TDF domains identified to BCTs	TDF, BCT Taxonomy (v1)
Development of a modified implementation intervention	Selection of intervention modifications to existing content	Delphi, APEASE
	Creation of modified intervention protocol	BCTs

## Results

### Step 1: qualitative interview study: identifying influences on Sepsis Six implementation

#### Participants

Nineteen nurses, 12 doctors, 2 midwives and 1 healthcare assistant (*n* = 34) participated in 3 focus groups and 17 individual interviews. Additional file 2: Table S2 shows the participants, their role and ward/department.

#### Influences on Sepsis Six implementation

All TDF domains except for “optimism” were identified in at least one transcript. The five most common domains identified in both focus group and individual interviews were social influences, knowledge, belief about consequences, memory/attention/decision-making and environmental context/resources. Twenty-one themes corresponding to 12 of the 14 TDF domains were identified as influencing implementation, either as barriers or levers. Of these, five (corresponding to seven TDF domains) were judged to be locally applicable when considering APEASE and informed the modified intervention. All transcript data coded by TDF domain and theme is shown in Additional file 3.

**Theme 1: knowing what to do and why**

**Domains:** ***knowledge, social/professional role and identity***

Knowledge about the triggers for sepsis and the Sepsis Six was seen to be a key influence on whether the bundle was implemented. Lack of familiarity with the bundle was mentioned by several respondents as a barrier:

…they seem to be - they are not aware how quick we have to - you know, to act on these kind of things, so that should be within one hour… (I14 general surgery nurse, line 45)

On the other hand, education about what to do and understanding that there was a clear and credible evidence base were seen as levers to successful implementation:

…the PARRT nurses are very well read on the subject…And to have somebody with the evidence to back up what they are advocating… And I think that after hearing why we are doing it everyone was much more inclined to do it. (I8 emergency department nurse, line 82)

Knowing what to do in situations that were not clear-cut was also mentioned as a barrier. For instance, before a definitive diagnosis of sepsis was made, it was often unclear whether symptoms such as high heart rate and fever were really due to sepsis.

… sometimes there might be a different reason for a patient to have, for instance a high heart rate and for it not to be related to sepsis. So that kind of grey area-where do you start them on the pathway because they’re triggering or do you take into account that they might be triggering for some other reason? (I1 emergency department senior nurse, line 49)

… originally it was “let’s hold off antibiotics we don’t know if there is an infective cause” whereas now that has pushed out of everyone’s mind. The thought is “Give them antibiotics, it’s not going to do them any harm” We can give them the first dose and that give us a bit more time to look at them as a patient, decide what is going on… (I4 emergency department nurse, line 86)

Health professionals working on inpatient wards also stated that patients often already had elements of the bundle administered and knowing what to do was more complicated in these cases.

A lot of the time things are already done, the oxygen is already on the fluids are already on… So it’s difficult to say when things actually start and when things are all done… (FG1 general surgery junior doctor, line 46)

**Theme 2: risks and benefits**

**Domain:** ***beliefs about consequences***

Belief and trust in the evidence of effectiveness of Sepsis Six was commonly reported as a lever to implementation.

And I think that it’s more established-the evidence base for Sepsis Six than perhaps for other protocols. It is effective. (FG1 general surgery junior doctor, line 208)

There was also a view amongst some participants that presentation of the “hard” facts had less of an impact on them than directly witnessing the improved condition of initially very sick patients.

I get more of a kick seeing the bundle work…looking after the patients and seeing them improve. So although it’s useful in some respects to know the figures …. I much prefer to see how the patient responds. (I5 emergency department nurse, line 44)

Fear of causing harm to the patient was often cited as preventing full implementation. This was commonly related to a concern about giving patients large volumes of intravenous fluids or broad-spectrum antibiotics without a confirmed diagnosis.

They didn’t prescribe fluids in time because they were worried about being overloaded. (I13 labour ward midwife, line 221)

However, it was evident that staff often chose to implement the bundle as they felt that the potential risks were outweighed by the benefits.

… it’s also knowing that the sepsis six does no harm. Obviously we won’t do it [un]necessarily…But it helps that there’s nothing harmful to the patient. (I9 medical assessment junior doctor, line 39)

**Theme 3: working together**

**Domains:** ***social influences, social/professional role and identity***

On the whole, cooperation between staff and working toward a common goal was seen as essential to implementation.

We’re part of team in A&E we feel that we can go to each other for help and support it definitely aids the running of it (Sepsis Six). (I6 emergency department nurse, line 135)

There was, however, a feeling that conflict between doctors and nurses led to problems:

I don’t really want to do that tradition of doctors versus nurses thing, but it was an issue for a while. I think because it (Sepsis Six) takes some of the power out of the doctors’ hands. (I5 emergency department nurse, line 76)

Working well together was also hindered by poor communication about patients both within and between teams.

There’s just a lack of communication about…you know… them triggering. And then you’ll get a call from the nurse saying that they are a bit unwell… but because you’re so busy you haven’t really thought about the triggers… and if they haven’t said “I Think that this is sepsis. They meet all the criteria, could you come urgently?” There might be a delay in getting to the patient. (I17 renal ward junior doctor, line 33).

**Theme 4: empowerment and support**

**Domains:** ***beliefs about capabilities, social/professional role and identity, behavioural regulation, social influences***

Participants spoke about the protocol giving them increased confidence in their abilities to carry out the six steps of the bundle and allowed them to challenge team members who were not implementing them.

…so next time that the nurses found themselves in that situation, where the doctors was going “Actually, well I don’t think we can do this”, they would go, “It’s fine, because it’s a part of the pathway. It’s all there in front of you”. (I10 emergency department assistant, line 84)

Despite this, there was still a feeling that challenging colleagues’ practice was not always easy.

So if it’s a junior or newly qualified nurse they might have difficulty going up to a senior [doctor] and saying that “Look [you’re] doing something wrong” (I1 emergency department senior nurse, line 86)

The introduction of the intervention in pilot areas as part of an iterative process in which versions were introduced, discussed with front-line staff and altered accordingly was seen as important in developing an intervention that was flexible, fit for purpose, developed professional practice and empowered nurses in particular.

…we’ve been able to work with (the Patient at Risk and Resuscitation team) to tell them what problems we’ve been having and to make the protocol a bit more flexible and to make it work for every patient rather than having problems with prescribers and their worries. (I4 emergency department nurse, line 54)

Participants also reported that support from and role-modelling by senior clinicians provided them with the confidence to implement.

Well if our seniors referred to it that would make a really big difference. So you see your seniors not starting sepsis six and you’re like “Well presumably I don’t need to start it then” (FG1 general surgery junior doctor, line 240)

**Theme 5: staffing levels**

**Domain:** ***environmental context and resources***

Several participants reported that staff shortages, especially at night, were likely to slow down implementation due to lack of doctors on duty and unclear communication that patients were septic, rather than non-specifically unwell, and therefore needed to be seen urgently.

Because if they become septic at night time it’s just harder to get doctors and everything just takes that extra bit longer. Because there’s less people. (FG3 general surgery nurse, line 77)

There’s just a lack of communication about..you know.. them triggering. And then you’ll get a call from the nurse saying that they are a bit unwell, they’re a bit tachycardic but because you’re so busy you haven’t really thought about the triggers (I17, renal ward junior doctor, line 33)

### Step 2: development of a modified intervention to improve Sepsis Six implementation

#### Round-table discussion and Delphi exercise

In the first round-table discussion, 26 potential intervention modifications were suggested, with a median APEASE score of 18.5 (IQR, 15.25–22.75) and range of 6 to the most positive score of 24 (see Table 2). Following the second round of the Delphi exercise, the median APEASE score was 19 (IQR, 15.75–21.75) with a range of 5 to 24. APEASE scores for 10 potential modifications increased between round 1 and round 2 and nine scores decreased. The median APEASE score for the modifications included in the final protocol was 21 (IQR, 19.5–23.5) compared with 18 (IQR, 14.5 19) for interventions not included in the final protocol. One relatively low scoring modification suggestion was included in the final intervention protocol following further group discussion. This was the inclusion of an instruction on how to perform a sepsis crash call to a doctor for assistance at night in the staff education component: after discussion, it was decided that this could be delivered easily in one short statement and would be acceptable and affordable. Three relatively highly scoring interventions: (a) the delivery of individual feedback for cases of non-implementation using a structured template, (b) delivery of feedback sessions to multi-disciplinary (e.g. nurses and doctors together) groups and (c) presentation of key journal references to staff were not included in the final protocol because after further group discussion, participants agreed that they would not be acceptable, practical or effective. Additional file 4: Table S4 shows the modification suggestions and APEASE scores for each Delphi round.

**Table 2:** Modifications to intervention based on themes and corresponding TDF domains identified in interviews

Theme	TDF Domains	Modification	BCTs delivered
New intervention component 1: partnership agreement			
Empowerment and support, working together	Belief about capabilities, social/pro role and identity, behavioural regulation, social influences	A collaborative partnership agreement between the intervention facilitator and clinical leads of the area is developed. This includes:	Behavioural contract^a^
Working together	Social influences, social professional role and identity	Details of when education and feedback sessions will be delivered and who will attend is decided by ward and facilitator collaboratively	Commitment,^a^ self-monitoring
Empowerment and support, working together	Belief about capabilities, social/pro role and identity, behavioural regulation, social influences	Two sepsis champions (1 doctor/1 nurse) nominated and supported	Social support (unspecified)
Empowerment and support	Belief about capabilities, social/professional role and identity, behavioural regulation, social influences	Commitment to Sepsis Six includes recognition of role model status and staff support	Identification of self as role-model,^a^ social support (unspecified)
Empowerment and support	Belief about capabilities, social/professional role and identity, behavioural regulation, social influences	Agreement that signatories will emphasise full group engagement with Sepsis Six	Commitment, behavioural contract
Empowerment and support, working together	Belief about capabilities, social/pro role and identity, behavioural regulation, social influences	Agreement that signatories will emphasise that challenging others is encouraged	Commitment, behavioural contract
Working together	Social influences, social professional role and identity	Agreement that staff attendance at training and feedback sessions will be recorded	Commitment, self-monitoring
Empowerment and support	Belief about capabilities, social/professional role and identity, behavioural regulation, social influences	Agreement on iterative nature of the document—all parties are involved it its creation and amendments	Commitment
Empowerment and support	Belief about capabilities, social/professional role and identity, behavioural regulation, social influences	Plan for collecting and sharing implementation data including details of who is responsible how it will be shared/displayed	Commitment, action planning^a^
Working together	Social influences, social/pro role and identity	Agreement that information shared at group feedback sessions will be cascaded down to all staff (those not able to attend feedback sessions)	Commitment
New intervention component 2: Hospital at Night Co-ordinator education			
Staffing levels	Environmental context and resources	Hospital at Night Co-ordinators receive sepsis education session including:	
Risks and benefits	Beliefs about consequences	Statement about the severity/health consequences of sepsis to patient	Information about health consequences
Risks and benefits	Beliefs about consequences	Statement about urgency: the consequences of not implementing within one hour	Information about health consequences
Risks and benefits	Beliefs about consequences	Statement about the importance of finding an on call doctor to attend triggering patients	Information about health consequences
Knowing what to do and why	Knowledge, social/pro role and identity	Statement of sepsis triggers	Instruction on how to perform a behaviour
Staffing levels	Environmental context and resources	“Sepsis bags” are made available in the PARRT office	Adding objects to environment
Working together	Social influences, social/pro role and identity	Instruction on how to perform a sepsis call to doctor	Instruction on how to perform a behaviour
Knowing what to do and why	Knowledge, social/pro role and identity	Statement about importance of using the six steps together as a bundle	Information about health consequences
Knowing what to do and why, risks and benefits	Knowledge, social/pro role and identity, beliefs about consequences	At least two “FAQs” are addressed, e.g. appropriate fluid volumes, evidence for oxygen administration, data on low C. Diff for broad spectrum abx, info about when hour begins, number needed to harm with bundle	Instruction on how to perform a behaviour, information about health consequences
Modifications to existing staff education component			
Empowerment and support	Belief about capabilities, social/professional role and identity, behavioural regulation, social influences	Statement that staff have authority to commence Sepsis Six using clinical discretion	Social support (unspecified)
Empowerment and support	Belief about capabilities, social/professional role and identity, behavioural regulation, social influences	Statement that full ward/department commitment is expected	Information about social consequences^a^
Empowerment and support, working together	Belief about capabilities, social/pro role and identity, behavioural regulation, social influences	Statement that challenging others should be un-personal and should be normalised	Generalisation of behaviour^a^
Risks and benefits	Beliefs about consequences	Evidence of patient outcomes presented quantitatively in at least two formats	Information about health consequences
Knowing what to do and why, working together	Knowledge, social/pro role and identity, social influences	Instruction on how to perform a sepsis call to doctor or Hospital at Night Co-ordinator	Instruction on how to perform a behaviour
Knowing what to do and why	Knowledge, social/pro role and identity	Statement about importance of implementing the six steps together as a bundle	Information about health consequences
Knowing what to do and why, risks and benefits	Knowledge, social/pro role and identity, beliefs about consequences	At least two “FAQs” are addressed, e.g. appropriate fluid volumes, evidence for oxygen administration, data on low C. Diff for broad spectrum abx, info about when hour begins, number needed to harm with bundle	Instruction on how to perform a behaviour, information about health consequences
Modifications to documents and materials component			
Staffing levels	Environmental context and resources	Sepsis bags made available to Hospital at Night Co-ordinators	Prompts/cues
Knowing what to do and why, risks and benefits	Knowledge, social/pro role and identity, beliefs about consequences	“FAQ” information sheet addresses: appropriate fluid volumes, evidence for oxygen administration, data on low C. Diff for broad spectrum abx, info about when hour begins, number needed to harm with bundle	Instruction on how to perform a behaviour

^a^New BCT (not used in original intervention)

#### Triangulation of findings from interviews, round-table discussion and Delphi exercise

Table 2 shows the agreed modifications and relevant BCTs linked to the themes and TDF domains. The modified intervention included two new components: a “partnership agreement” contract between the Patient Safety Facilitator and the relevant ward or department’s clinical lead(s) and a Sepsis Six education programme for the Hospital at Night Co-ordinators, a role that involves carrying out clinical tasks traditionally carried out by junior doctors (patient assessment/triage, cannulation, etc.) if a patient’s condition deteriorates overnight. The Night Co-ordinator is the first point of call for a medical emergency when a doctor is not on site out of hours. Additionally, modifications to two of the existing components, staff education sessions and documents and materials were made.

The partnership agreement was introduced in light of comments regarding senior staff involvement and modelling and the effectiveness of the collaborative approach already in use. The mutual agreement was a behavioural contract that stipulated the type and extent of responsibility of the clinical lead(s) for implementation. For instance, it outlined the degree to which the clinical lead(s)/Patient Safety Facilitator would be involved with data collection or delivery of feedback to staff. It was developed in order to be flexible enough for implementation in a range of settings and endorsed engagement of all clinical staff with team members being encouraged to challenge one another other when the bundle was not implemented.

The Hospital at Night Co-ordinators, who had not previously received any Sepsis Six education, were to receive a programme to educate and empower them to diagnose sepsis, initiate the bundle and summon an on call doctor to attend a medical emergency. This addressed barriers to implementation posed by a shortage of doctors at night.

The existing staff education programme was modified to address fears about harming patients (e.g. with intravenous fluid), emphasise the effectiveness of Sepsis Six, improve knowledge of what to do and why, normalise challenging colleagues and empower nurses to initiate the bundle and call the Night Co-ordinator or on-call doctor. There were some modifications made to documents and materials including the inclusion of an FAQ document that addressed knowledge deficits and providing sepsis bags containing all instruments needed for implementation for Night Co-ordinators.

A summary of the final modified intervention detailing each component, to whom and how it was delivered, and the frequency with which it was delivered (i.e. according to the Template for Intervention Description and Replication; TIDieR) [36] is shown in Table 3. The modifications consisted of 12 BCTs, six of which were not used in the original intervention. These were: *behavioural contract*, *commitment*, *action planning*, *identification of role*-*models*, *information about social consequences of behaviour*, and *generalisation of target behaviour*. See Additional file 5 for a full intervention protocol including the aims, BCTs, and mode and dose of delivery of each.

**Table 3:** Modified Sepsis Six intervention summary based on Template for Intervention Development and Replication (TIDier)

Modified intervention components	Rationale	Mode of delivery	Delivered to	When/how often
Sepsis Six introductory education sessions including target setting of 95 % implementation	To familiarise staff with the bundle and generate enthusiasm	Face to face (group)	Doctors and nurses	Once when Sepsis Six is first introduced and once at each new/junior staff induction to the ward
Promotional and educational documents^a^	To educate staff about the pathway and promote self-monitoring	Documents	Doctors and nurses	Ongoing
Materials provided to aid implementation^b^	To make implementation more convenient	Environment changes	Resources varied between wards	Ongoing
New intervention components				
Partnership agreement contract between clinical leads in area and intervention facilitator	To engage senior staff members, highlight role model status and ensure collective commitment to implementation	Documents	Clinical lead in intervention area and nurse facilitator	Once when “Sepsis Six” is first introduced to area
Hospital at Night Co-ordinator education	To stimulate action towards aiding staff with implementation on night shifts	Face-to-face (individual)	Hospital at Night Co-ordinators	Once
Unmodified/original intervention components				
Training (septic patient simulation)	To train staff on how to implement	Face-to-face (group)	Minority of doctors and nurses (ad hoc)	Ad hoc, approximately bi-monthly
Data measurement and group feedback- daily implementation rates displayed in staff break area and verbal feedback given	To focus staff on targets and progress	Rates displayed, feedback delivered face-to-face (group)	All available doctors and nurses (majority nurses) on shift	Rates displayed daily, weekly or bi-weekly feedback sessions
Individual personalised feedback for staff involved in incidents when bundle was not fully implemented	To target specific incidents of non-compliance	Face to face (group)	Staff involved in incidents where bundle was not correctly or fully implemented	Ad hoc, ~2 staff per week

## Discussion

The study is a systematic, theoretically guided approach to modification of an existing patient safety programme and illustrates how theory can be used to enhance a pragmatically developed intervention using P-D-S-A cycles. From interviews with health professionals, five themes were identified as acting as barriers or levers to implementation of the Sepsis Six clinical care bundle. These were used to identify gaps in the existing intervention and informed modifications. Two new intervention components were added: a partnership agreement (targeting increased clarity of roles and responsibilities and support from senior staff and normalising challenging colleagues’ clinical decision-making) and the development of training for Hospital at Night Co-ordinators (addressing potential shortfalls in implementation of the bundle at night). In addition, modifications were made to existing staff education sessions and educational/promotional documentation, targeting knowledge deficits, belief about consequences of implementing and improving accessibility of resources.

Much of the published quality improvement work has used more pragmatic approaches, without the explicit use of theory [8, 12]. Both the TDF and BCT Taxonomy (v1), however, are becoming increasingly popular for understanding clinical behaviour and guiding implementation intervention design “from scratch”. Examples of published work using the TDF and BCT Taxonomy to guide quality improvement are seen in antibiotic prescribing [30] and organ donation [31], but to the authors’ knowledge, they have not yet been used in the design of interventions to improve implementation sepsis guidelines. In addition, as far as we are aware, this is the first example of a study that has used these tools to “enhance” an existing pragmatically developed intervention. This approach more accurately reflects the real world of clinical practice where interventions, which have achieved some level of success, are in place, but are not fully reported and may require improvement.

The use of feedback interventions and targeting of hospital at night teams is supported by published reports of other quality improvement programmes. For example, audit of health professional behaviour combined with feedback is a widely used strategy for improving professional practice [37]. Investigation of collaborative work between critical care nursing and hospital at night teams has demonstrated significant impact on patient mortality [38]. In contrast, although behavioural contracts have been used between patients and their healthcare practitioner, for example for promoting medication adherence [39], to our knowledge, their use in quality improvement programmes from between health professionals is novel.

This study demonstrates how a synthesis can be achieved between a pragmatic intervention and a theoretically guided approach to its enhancement. The P-D-S-A cycle approach used at the study hospital was flexible and practical, allowing for changes to be introduced and discarded incrementally with input from those receiving the intervention, allowing for adaptation to different contexts and clinical areas that faced unique implementation challenges [20]. This was identified as an important lever to implementation by participants. However, this process did not define the intervention exactly nor did it target all potential mechanisms of action.

Combining this approach with tools developed by behavioural scientists allowed us to do several things. First, using the TDF to structure the interview guide and code responses provided a coherent framework for thematic analysis of influences on implementation. These data combined with a description of the intervention from a previous stage in the research [16] enabled comprehensive thinking about existing practice, including identification of what might be missing and provided an explicit rationale for modification. Second, the BCT Taxonomy and published guidance on mapping this to TDF domains [23] allowed us to highlight potential intervention strategies that had not already been used and ensured that modifications targeted the barriers and enhanced the levers identified. Third, we were able to use the BCT Taxonomy to comprehensively report the intervention’s “active ingredients” using a shared language. This is in keeping with the increased emphasis on a transparent and robust rationale for the selection of intervention content and comprehensive reporting [36, 40].

The collaborative approach to modifying the intervention is an important strength of this work, especially given the identification of this as a significant lever to the success of the intervention. A wide range of clinical staff from all pilot areas were involved from the start in identifying barriers and levers. The nursing team responsible for facilitating the intervention were then closely involved in modifying the intervention in a way that was consistent with theory, compatible with the widely used P-D-S-A cycle and was judged to be practical and feasible.

The feasibility and outcome of any quality improvement programme is always a product of content, context and application [41]. The main limitation of this study is that it was conducted in a single hospital in clinical areas that were already implementing the bundle. We cannot draw conclusions about generalisation to other settings. Secondly, although it was originally intended to test fidelity to intervention and effectiveness, it was not possible to do this in the time available since roll-out to other wards was postponed. Nevertheless, although we cannot say that the modified intervention was better than the original at this stage, it has now been rolled out to other trust areas and evaluation is planned as a final stage of the research. Additionally, it is not known whether this work would have been achievable without the input of those with experience using the TDF and BCT Taxonomy and/or time allocated to learn how to apply them. The most time-consuming element of the process, however, was organising, carrying out, transcribing and coding of the interviews. Although it was not formally analysed, the fact that the most commonly mentioned TDF domains were the same in focus groups and individual interviews suggests that using focus groups alone may provide valid data. This may provide an acceptable alternative to the use of individual interviews in contexts where researcher time and resources are limited.

Further work should aim to implement this intervention protocol in different settings and evaluate its feasibility, fidelity and effectiveness including the practicality of continuing P-D-S-A cycles and reporting further changes. In addition, the transferability of the TDF and BCT Taxonomy (v1) for enhancing other pragmatically designed interventions in different contexts and for different behaviours should be explored.

## Conclusions

This study demonstrates the feasibility of using the TDF and BCT Taxonomy (v1) to modify existing, pragmatically designed implementation interventions. As such, it shows how we might bridge the gap between behavioural theory to design interventions and commonly used pragmatic approaches to quality improvement, such as the P-D-S-A cycle. Combining these two approaches could act as a model for future work to enhance existing interventions in a way that is transparent, replicable, theoretically guided and practical.

## Additional files

Additional file 1: **Focus group/interview topic guide.** (DOCX 46 kb)

Additional file 2: Table S2. Interview/focus group participants for step 1, qualitative interview study. (DOCX 16 kb)

Additional file 3: **Interview data TDF coding and thematic analysis.** (XLSX 98 kb)

Additional file 4: Table S4. Results from Delphi exercise—modification suggestions and APEASE scores (out of 24 for 4 scorers). (DOCX 27 kb)

Additional file 5: **“Sepsis Six” modified intervention protocol.** (DOCX 47 kb)

## Abbreviations

BCT: behaviour change technique
NHS: National Health Service
TDF: Theoretical Domains Framework
TIDieR: Template for Intervention Description and Replication
