# Supplementary material for: Bridging the gap between pragmatic intervention design and theory: using behavioural science tools to modify an existing quality improvement programme to implement “Sepsis Six”
Source: Implement Sci. 2016 Feb 3;11:14. doi: 10.1186/s13012-016-0376-8 (PMC4739425; doi:10.1186/s13012-016-0376-8)
Supplement: Supplementary file 1 — Focus group/interview topic guide. (DOCX 46 kb) [file 13012_2016_376_MOESM1_ESM.docx]

**Focus Group/Interview Topic Guide**

Thanks for agreeing to participate in this interview/focus group.

The general aim of the study is to help us understand more about how the ‘Sepsis Six’ has been implemented here at the Royal Free. We know that Sepsis Six often gets implemented according to protocol, but sometimes it doesn’t and we want to explore the factors that might influence this. This will help the sepsis nursing team with implementing the bundle better in other wards. Some of these questions might seem obvious or strange, but your ideas will help us to develop ‘Sepsis Six’ further and improve patient outcomes.

I am going to record us if that’s okay with you just to make sure we capture everything you say. The only people who will hear the interviews are researchers at UCL – so no-one at the Royal Free. We will publish a synthesis of all the information we collect, but any identifying information will be removed from the transcripts. Also if you change your mind at any time and don’t want what you say to be used, you can let us know and we will destroy the tape. How does that sound?

**Introductory Questions**

- What is your role and how long you have been using the Sepsis Six bundle in your job.
- What efforts have you noticed to promote Sepsis Six at Royal Free? What do you think has worked or not worked? Why? What have people found most valuable?
- Okay, let’s generate some typical incident(s) where Sepsis Six didn’t go according to protocol. Can you talk me through what happens? It may be helpful to think about specific times.

*Interviewees generate some typical ‘types’ of variation protocol.*

Eg- *Drs are reticent to give fluids quickly so the bundle takes longer than an hour*, *new members of staff come on the ward and they are unfamiliar with the bundle and implementation rates dip,* etc.

- Which of these situations are most frequent or typical?
- (After deciding this). Okay let’s focus on ‘A’ situation and I’m going to ask some more specific questions. When answering, try and think about your experience and perceptions of a specific ‘A’ situation.
- Why do you think things didn’t go according to protocol?
- What do you think might be done differently in future to help Sepsis Six get implemented according to protocol? What would have helped?
- I’m going to ask some more specific questions about what you think might be the factors that are influencing implementation here. For this, it may be helpful to think about ‘Sepsis Six’ being done according to protocol as a set of behaviours. It isn’t just one thing that needs to be done, in fact it is a series of behaviours or tasks that need to be completed in order to have implementation.

*Plus TDF prompts/questions below when required/mentioned by interviewee

**Semi-structured interview/focus group topic guide**

The schedule is informed by initial ethnographic/field notes of observations of bundle implementation on wards and conversations with health professionals using the bundle and the 14 domains of the Theoretical Domains Framework (TDF; Cane et al. 2012) and components of the COM-B model (Michie et al. 2011).

**Note** The questions below can be formed around these ‘typical circumstances’ of protocol variation that participant has generated above…e.g*. ‘Is remembering the steps of Sepsis Six ever an issue?’*

becomes *‘in this circumstance you mentioned, is remembering steps ever an issue?’* etc.

| COM-B | TDF | Topic question |
| --- | --- | --- |
| Psychological capability | Memory, attention and decision processes | Is remembering the steps of Sepsis Six ever an issue?  What would help people remember it? |
|  | Behavioural Regulation | Do you think it would be helpful to have additional systems in place such as people monitoring themselves or action planning? |
| Psychological capability | Knowledge | Would people having more knowledge of sepsis and the Sepsis Six bundle make it more likely to be implemented according to protocol? Which specific steps if any would they need more knowledge about? |
| Physical Capability | Skills | Are there any additional skills you and your colleagues could acquire that would make your practice more likely to go according to protocol? |
|  | Beliefs about capabilities | Do you think staff having confidence in their abilities is important for implementing Sepsis Six?  How confident are you and your colleagues in your abilities to keep to the Sepsis Six protocol? What could be done to increase your or their confidence? |
| Physical opportunity | Environmental context & resources | Does anything in the environment ever affect whether Sepsis Six goes according to protocol (by giving people more of an opportunity to carry out the behaviours)?  Is there anything that could be changed in the working environment that would help Sepsis Six go according to protocol? |
| Social opportunity | Social influences | Do you think people around you/the social environment ever affects implementation? If so, in what way?  *Prompt on social support, social norms, conflict and conformity here.*  What do you think could be done to change this for the better? |
| Reflective motivation | Social/professional role & identity | Is there anything about being part of a professional group/organisation that influences Sepsis Six implementation? I’m thinking about things like professional identity, professional boundaries, and hierarchical structure.  Do you think people ever just don’t think certain behaviours should be part of their role? (e.g. Dr’s don’t do …..)  What changes could be made (to the above) to improve it? |
| Reflective motivation | Beliefs about consequences | How much difference do you think your colleagues believe implementing the Sepsis Six bundle makes? Do people believe in the evidence? If it makes a difference, who does it make a difference to?  Do you think it would help people if they knew more about the consequences of carrying out each behaviour within the hour? (consequences for patient/self/colleagues). Is this important for making sure it get implemented? |
| Reflective motivation | Intention | Do you think people always intend to implement Sepsis Six when it is appropriate? There are always competing priorities at work. Do you think implementing Sepsis Six is a priority? Are there times when it is not a priority/becomes less of a priority? Do other things get in the way?  What would help it to be more of a priority for staff in these circumstances? |
|  | Goals | Does having targets to meet and goals around doing Sepsis Six help or hinder implementation? |
| Automatic motivation | Reinforcement | Usually when bundles are not implemented, it is for a reason. Are there sometimes negative consequences for sticking to the Sepsis Six protocol? If so, what are they? For whom? Could this be changed? How? |
|  | Optimism | Do you think people whether feel optimistic or pessimistic about Sepsis Six influences implementation?  Do you think it would help if people felt more optimistic about the bundle? |
| Automatic motivation | Emotion | Do you think that emotional states get in the way of implementation?  Are there any of the specific behaviours here that are part of Sepsis Six that are influenced by emotional state?  What would help people feel ‘better’ about implementing? |

**Coding Guidelines for TDF Analysis of Interview/Focus Group Transcripts**

**General notes on coding**

- Utterances can be coded according to more than one domain.
- Utterances should be coded as ‘influences’ rather than barriers and levers. If participant mentions Domain x influences or would influence implementation it is coded.

**Domain Coding**

**Knowledge:**

Interviewee mentions barrier or lever that relates to:

- Not knowing about: sepsis- what it is, the Sepsis Six or any of the steps, or the triggers for Sepsis Six, e.g. how to identify it.
- Knowledge of whether or not patient is septic. This includes not believing patient is septic despite triggering, e.g. ‘they look well’. If insecurity over whether patient is septic and therefore conscious decision is made not to commence Sepsis Six/to delay it is mentioned code also as **intention.**
- Factors that mimic sepsis creating confusion/false triggers that are not sepsis
- Knowledge of the evidence-base for giving Sepsis Six or any one of the steps, e.g. ‘ knowing why we are doing a certain part of Sepsis Six’.
- Knowledge of whether entire bundle is needed because patient has already had some/one of the steps (e.g. fluids, oxygen) for other clinical reason.
- Procedural knowledge- covers all areas around ‘knowing what to do’ when someone triggers. Knowing the protocol, the procedures to take including how to carry out all steps.
- Do not code if ‘education’ is mentioned but not elaborated on how the education affected implementation – if it was knowledge enhancing, then yes.
- Retriggering creating confusion, i.e. knowledge of whether to re-implement any steps

**Skills:**

Participant mentions barrier or lever that relates to:

- Being unable or able/trained to carry out a step of the Sepsis Six (e.g. some midwives have not been trained on how to use the blood-gas machine
- Clinical proficiency, when experience or lack thereof is mentioned (e.g. with recognition of sepsis/diagnosis or recognition of something else that mimics sepsis)
- Behavioural practice of Sepsis Six because improves competency and skills, note if practice gets people ‘used to it’ or doing it by habit, code also as **behavioural regulation.** E.g ‘we have done it so many times now, we are used to it’.
- Note code as **Skills** if referring to staff ‘being able to/not being able to or trained to’ carry out a task related to Sepsis Six If they could be trained to do so, but personally do not have the current skills and THIS is the influence on implementation (skills or lack of skills to do a task then code as **skills.** If staff also are not allowed to carry out task because it is not part of their role or their duties as a nurse/EDA/midwife etc., e.g. prescribing antibiotics and this is also influencing implementation, code also as **Social/Professional Role & Identity**. If it is only that it is not part of their role to do a certain job, then code only as Social/Pro Role & Identity. In other words, code the domain(s) that is the barrier or lever here.

**Memory/Attention/Decision Making (MAD):**

Participant mentions barrier or lever that relates to:

- Inability to focus attention on or keep Sepsis Six in mind (e.g. due to busyness)
- Forgetting to carry out Sepsis Six
- Forgetting a step of the Sepsis Six
- Note that **MAD** domain should refer to automatic, not reflective processes (Psychological Capability), If ‘decision’ to carry out or not carry out is intentional/reflective due to competing priorities, code as **intention**.
- Note that if forgetting or inability to focus is due to busyness on wards, also code **environmental context and resources**.
- Note resources (i.e. Sepsis Bags, Trolley or paper protocol) and ‘checklists’ may be coded as **MAD** if interviewee mentions that they perform the function of ‘reminding’ or ‘prompting’ Sepsis Six steps or triggers. If checklist is used as monitoring behaviour, code as **behavioural regulation.**
- Code as MAD when interviewee uses language of ‘remember’, ‘prompt’, ‘remind’, ‘forget’, ‘memory’.

**Behavioural Regulation:**

Participant mentions barrier or lever that relates to:

- Self-monitoring (writing down steps and timing) Sepsis Six completion, e.g. using checklists and stickers, Sepsis Cross which monitors implementation real time.
- Specific mention of any system or processes that help things go according to protocol
- Specific mention of habit or habit formation and/or if language of ‘used to it’, ‘we do it automatically’, ‘don’t think about it anymore’ ‘just knowing what to do straight away’ is used by interviewee. E.g. the gap between intention and behaviour is moderated.
- Reciprocal feedback system or HCWs being part of the pathway development and amendments. i.e. protocol not currently working, trust monitors not great outcomes, asks why, staff feedback and make suggestions, this is reviewed, amendments made to protocol.
- Note reciprocal feedback is coded as **Behavioural Regulation** as it is a system which facilitates implementation of the bundle, as **Social Pro Role/ID** because HCWs are developing their role and taking ownership of the bundle and as **Social Influences** as it is a collaborative process between PARRT and staff. This is also often, but not always coded as **beliefs about capabilities** if improving professional confidence and self-efficacy, perceived behavioural control over the protocol is mentioned.
- Note if barrier or lever relates to a system of communication of where on the pathway the patient is/whether patient has been put on Sepsis Six, code as **behavioural regulation** and also **social influences** (communication).
- Note code ‘escalation’ system as **social influences**, social support/communication about patient and not behavioural regulation
- Note feedback itself is not coded necessarily coded as **behavioural regulation**. If feedback is mentioned as influencing implementation, code as the function that the feedback serves, i.e. is the feedback useful because it is providing knowledge of Sepsis Six? (code **knowledge**) ,reminding of six steps? (code **MAD**), giving criticism/reprimanding? (code **beliefs about consequences)**, empowering individuals to take ownership of pathway and/or have professional confidence through reciprocal feedback (code **behavioural regulation** ,**beliefs about capabilities** and **Social/Pro role/ID** as detailed above)

**Social Influences:**

Participant mentions barrier or lever that relates to:

- Other people affecting whether or not Sepsis Six is implemented.
- Escalating issues with implementation to higher levels or the PARRT team
- Commitment to the organisation is **social influences**, note this is not **Social Pro Role and ID** because it is about OTHERS, not the self.
- Social support, but not practical staffing support/human resources
- Note if support is practical, i.e. other staff helping with implementation, this is not social support and should be coded as **Environmental Context and Resources**.
- Superiors or peers influencing whether he/she carries it out, i.e. social norms and modelling. e.g. there is a delay in prescription, because prescribing the wrong antibiotic would frowned upon by other professionals
- Communication, verbal and written communication, i.e. on documents, CAS cards, patient notes, stickers, checklist protocols affects implementation- not just mere mention of comms
- Conflict, cooperation, teamwork between teams and individual, note that conflict or cooperation that relates specifically to ingroup/outgroup identity is also coded as **Social Pro Role & ID** (e.g. doctors and nurses or nurses and midwives, etc. should also be coded as
- Note reciprocal feedback is coded as **Behavioural regulation** as it is a system which facilitates implementation of the bundle, as **Social Pro Role/ID** because HCWs are developing their role and taking ownership of the bundle and as **Social Influences** as it is a collaborative process between PARRT and staff. This is also often, but not always coded as **beliefs about capabilities** if improving professional confidence and self-efficacy, perceived behavioural control over the protocol is mentioned.
- Believing that Sepsis Six has already been carried out by others (teams, individuals, wards)
- Note if barrier or lever relates to communication of where on the pathway the patient is/whether patient has been put on the pathway, code as **social influences** and also **behavioural regulation** as this relates to system of monitoring and communicating.
- Conflict, power, authority and hierarchy
- Organisational culture influencing implementation, i.e. others working hard/caring or not caring, not being interested in the Sepsis Six getting done
- If protocol is mentioned as people ‘must do’ or is ‘expected’ of them by trust, as part of their role code as **social influences** as this is behaviour being influenced by others. Note that if protocol prompts memory, code as **MAD** if it educates people about what to do, also code as **Knowledge**.
- Note if conflict or cooperation is specifically mentioned as being a result of professional group identity (e.g. doctors vs. nurses), code also as **social/professional role & identity**.

**Social/Professional Role & Identity (S/P ID):**

Participant mentions barrier or lever that relates to:

- Relates to one’s own SELF-IDENTITY. **SP Role & ID** is more centred on the individual whereas **social influences** relates to those around you influencing behaviour.
- Sepsis Six being/not being someone’s responsibility or job because of who they are or what their role is
- Following a protocol, or specifically following Sepsis Six fitting or not fitting with a person’s personal or professional identity, e.g. if someone mentions ‘As I doctor, I use clinical judgement over protocol’, not wanting to be seen as someone who just follows protocol
- Mention of clinical judgement alone is not enough to code as **SP Role & ID**.
- Role development, e.g. the use of protocol is thought to have developed a person’s role or idea of self as a professional, empowerment
- Reciprocal feedback system or HCWs being part of the pathway development and amendments. i.e. protocol not currently working, trust monitors not great outcomes, asks why, staff feedback and make suggestions, this is reviewed, amendments made to protocol.
- Note reciprocal feedback is coded as Behavioural Regulation as it is a system which facilitates implementation of the bundle, as Social Pro Role/ID because HCWs are developing their role and taking ownership of the bundle and as Social Influences as it is a collaborative process between PARRT and staff. This is also often, but not always coded as beliefs about capabilities if improving professional confidence and self-efficacy, perceived behavioural control over the protocol is mentioned.
- Note if staff do not carry out task because it is not part of their role, e.g. prescribing antibiotics, code as **S/P ID** and this is affecting implementation. Code as **Skills** if referring to staff ‘being able to/not being able to or trained to’ carry out a task related to Sepsis Six affecting implementation. In other words if staff member is unable to do a task but could be trained to do so as part of implementation intervention and this slows down implementation, this is coded as **skills** as opposed to certain roles preventing one from carrying out a step of Sepsis Six- then code as **S/P ID.** If both that it is not part of role and not able to, code both **skills** and **S/P ID**. Note cod the domain(s) that influences implementation here.
- Conflict or cooperation that relates specifically to ingroup/outgroup identity (e.g. doctors and nurses or nurses and midwives, etc. should also be coded as **social influences**
- If staff are not allowed to carry out task because it is not part of their role, e.g. prescribing antibiotics, code as **Social/Professional Role & Identity** and **skills**. If they only cannot do it due to not being trained or being unable, but could learn to/be trained to, code only as **skills.**

**Environmental Context and Resources:**

Participant mentions barrier or lever that relates to:

- Materials and resources being available/unavailable, able to find, in the same place
- Staff shortages or time of day (e.g. nights being a barrier)
- Time pressure as an environmental stressor
- Busyness
- Note that busyness is followed by forgetting or not thinking about sepsis or a patient code also **MAD**
- Note that if busyness is followed by a reflective decision not to carry out Sepsis Six or to wait to carry out because of competing priorities (other patients to attend to first), code also **intention.**
- Note if resources (i.e. sepsis trolley or bags) also prompts or reminds, code also as MAD. If only works as a prompt, code only **MAD**. If resources are being used for self-monitoring tools or checklists for each step, code as **behavioural regulation**.

**Beliefs about Consequences:**

Participant mentions barrier or lever that relates to:

- Any mention of what a person believes will or will not happen if Sepsis Six, or any step of the Sepsis Six is done according to protocol or NOT done according protocol.
- **To patient**

1. Anticipated regret for harming patient, e.g. chronically ill patients may be harmed by giving fluids stats or oxygen, prescribing wrong medication and harming patient
2. Believing or not believing that the Sepsis Six (or any element of the six) will help patient clinically. This may be due to 1. Believing/not believing patient is septic therefore not correct clinical route, i.e. looks well or has a viral infection, etc. This is also coded as **knowledge** E as HCW does not know whether patient is septic or 2. Believing/not believing in the evidence base for improving patient outcomes
3. Note if HCW believes that Sepsis Six will help, but believes another clinical consideration (competing priorities) is more important or will have graver consequences at the time code as **beliefs about consequences** and also **intention.**

**Beliefs about capabilities:**

Participant mentions barrier or lever that relates to:

- Confidence in carrying out all steps of the Sepsis Six, e.g. believing it is easy to do
- Self-esteem
- Professional confidence or self-belief
- Perceived behavioural control
- Empowerment, i.e. the bundle itself makes nurse feels empowered or boosts professional confidence
- Reciprocal feedback loop such that HCWs are involved in creation and amendments to the protocol if professional confidence perceived behavioural control, self-efficacy, empowerment are mentioned.
- Note reciprocal feedback is always also coded as **behavioural regulation** and **Social Pro Role/ID**

**Intention:**

Participant mentions barrier or lever that relates to:

- Global or general intention to carry out Sepsis Six- think stages of change model and a feeling that Sepsis Six is in general a priority that staff are or are not committed to.
- Any mention of competing priorities or balancing what needs to be done when
- Any explicit mention of reflective decision to implement or not implement the Sepsis Six or any one of the steps despite the ‘evidence’. Also may need to code the reason for deciding that, e.g. beliefs about consequences. For example, if patient is triggering, but the HCW says he/she does not want to implement yet because unsure of source, should be coded as INTENTION and KNOWLEDGE. If also mentioned that antibiotics could harm patient, code also as BELIEFS ABOUT CONSEQUENCES.
- Not wanting to or choosing not to carry out Sepsis Six because of other tasks/clinical considerations or patients needing attention are deemed more important. Can also apply to the same patient- other clinical considerations.
- Note if decision making is impaired (i.e. focus and attention to decision impaired) code as **MAD.**
- Note if interviewee mentions not ‘wanting’ to carry out Sepsis Six code, this is not enough to code as **intention.** Must code reason for not wanting to. For example, ‘Doctors don’t want to do Sepsis Six on Renal patients because they are worried they will fluid overload them and hurt them’ is coded as **beliefs about consequences**.
- If insecurity over whether patient is septic and therefore conscious decision is made not to commence Sepsis Six/to delay it is mentioned code a as **intention** and **knowledge.** Also may need to code the reason for deciding not to, e.g. **beliefs about consequences.**

**Goals:**

Participant mentions barrier or lever that relates to:

- Having targets or goals to reach, i.e. 95% implementation helps implementation
- Note code only when interviewee mentions concrete goals or target, if ‘wanting’ or ‘intending’ code **intention**.
- Note the words ‘aim to’ or ‘it is my goal to’ is not enough to code **goals.** Only code as **goals** when actual goal-setting, targets are referred to as influencing implementation.

**Optimism:**

Participant mentions barrier or lever that relates to:

- Belief that Sepsis Six will or will not go according to protocol
- Unrealistic optimism, e.g. everything will be fine and we don’t have to work hard to reach targets.
- General mind-set about whether Sepsis Six targets will be met- an optimistic or pessimistic state.

**Emotion:**

Participant mentions barrier or lever that relates to:

- Specific mention that carrying out Sepsis Six evokes an emotion, e.g. pride, anxiety, feeling good about oneself, fear, tiredness, stress, burnout.

**Reinforcement:**

Participant mentions barrier or lever that relates to:

- Reward, incentive or punishment contingent upon reaching targets
- Reward, incentive or punishment contingent upon progress toward target, e.g. going from 80-90% in one month
- Social reward, for example praise, congratulations is coded as **reinforcement.**
- Negative feedback for incidence of poor implementation, e.g. staff anticipate receiving this feedback if they don’t implement according to protocol and this affects implementation.
- Punishment or reprimanding for not reaching target or for dipping down in implementation.
