# Supplementary material for: Bridging the gap between pragmatic intervention design and theory: using behavioural science tools to modify an existing quality improvement programme to implement “Sepsis Six”
Source: Implement Sci. 2016 Feb 3;11:14. doi: 10.1186/s13012-016-0376-8 (PMC4739425; doi:10.1186/s13012-016-0376-8)
Supplement: Supplementary file 2 — Interview/focus group participants for step 1, qualitative interview study. (DOCX 16 kb) [file 13012_2016_376_MOESM2_ESM.docx]

| **Transcript** | **Role(s)** | **Ward or department** |
| --- | --- | --- |
| Focus Group 1 | Foundation Year 1 junior doctors (*n=*5) | General surgery |
| Focus Group 2 | Foundation Year 2 junior doctors (*n=*2) | Emergency department |
| Focus Group 3 | Nurses (*n=*10) | General surgery |
| Interview 1 | Senior nurse | Emergency department |
| Interview 2 | Consultant | Emergency department |
| Interview 3 | Nurse | Emergency department |
| Interview 4 | Nurse | Emergency department |
| Interview 5 | Nurse | Emergency department |
| Interview 6 | Nurse | Emergency department |
| Interview 7 | Nurse | Emergency department |
| Interview 8 | Nurse | Emergency department |
| Interview 9 | Foundation Year 1 junior doctor | Medical assessment |
| Interview 10 | Emergency department assistant | Emergency department |
| Interview 11 | Matron | Emergency department |
| Interview 12 | Midwife | Labour ward |
| Interview 13 | Midwife | Labour ward |
| Interview 14 | Nurse | General surgery |
| Interview 15 | Registrar doctor | Emergency department |
| Interview 16 | Registrar doctor | Simulation Centre |
| Interview 17 | Foundation Year 1 junior doctor | Renal ward |

Table S2: Interview/Focus Group participants for Step 1, Qualitative Interview Study
