# Supplementary material for: Bridging the gap between pragmatic intervention design and theory: using behavioural science tools to modify an existing quality improvement programme to implement “Sepsis Six”
Source: Implement Sci. 2016 Feb 3;11:14. doi: 10.1186/s13012-016-0376-8 (PMC4739425; doi:10.1186/s13012-016-0376-8)
Supplement: Supplementary file 4 — Results from Delphi exercise—modification suggestions and APEASE scores (out of 24 for 4 scorers). (DOCX 27 kb) [file 13012_2016_376_MOESM4_ESM.docx]

**Table S4:** Results from Delphi exercise- modification suggestions and APEASE scores (out of 24 for 4 scorers)

|  | **Delphi Round 1** | | | | | | | **Delphi Round 2** | | | | | | |
| --- | --- | --- | --- | --- | --- | --- | --- | --- | --- | --- | --- | --- | --- | --- |
| **Potential Modification Suggestion** | **A** | **P** | **E** | **A** | **S** | **E** | **Sum** | **A** | **P** | **E** | **A** | **S** | **E** | **Sum** |
| Two interventions run concurrently (different programme for areas with low vs high sepsis cases) | 4 | 4 | 4 | 4 | 2 | 2 | **20** | 4 | 3 | 3 | 4 | 3 | 2 | **19** |
| Partnership agreement between sepsis team and ward/ department is drawn up | 4 | 2 | 3 | 3 | 4 | 3 | **19** | 4 | 4 | 4 | 3 | 4 | 4 | **23** |
| Middle grade registrars are appointed as ‘Sepsis Champions’ to drive improvement work | 2 | 1 | 3 | 3 | 2 | 3 | **14** | 3 | 2 | 4 | 3 | 3 | 4 | **19** |
| Two ‘sepsis champions’ are appointed per ward/department (ideally one Dr and one nurse or midwife) | 4 | 4 | 4 | 4 | 4 | 4 | **24** | 3 | 2 | 4 | 3 | 4 | 4 | **20** |
| Education, training and feedback sessions are delivered by sepsis champions in areas with low sepsis cases | 3 | 2 | 2 | 2 | 2 | 2 | **13** | 2 | 1 | 1 | 1 | 2 | 2 | **9** |
| Sepsis champions deliver education sessions to new staff | 4 | 4 | 3 | 3 | 3 | 3 | **20** | 3 | 3 | 2 | 3 | 2 | 2 | **15** |
| In education sessions, statement that full are commitment to the guidelines is expected is given | 4 | 4 | 4 | 4 | 4 | 4 | **24** | 4 | 4 | 4 | 4 | 4 | 4 | **24** |
| In education sessions, statement that challenging colleagues is un-personal and should be normalised is given | 4 | 4 | 4 | 3 | 4 | 4 | **23** | 4 | 4 | 4 | 3 | 4 | 4 | **23** |
| In education sessions, evidence for effectiveness of the guidelines presented in at least two quantitative formats (i.e. number needed to treat, % lives saved, mortality and length of stay numbers) | 3 | 3 | 3 | 3 | 3 | 3 | **18** | 3 | 3 | 4 | 3 | 4 | 4 | **21** |
| Education sessions address at least two ‘Frequently Asked Questions’ (FAQs) about the guidelines | 4 | 4 | 4 | 4 | 4 | 4 | **24** | 4 | 4 | 4 | 4 | 4 | 4 | **24** |
| Individual feedback is delivered using structured template | 3 | 3 | 4 | 4 | 4 | 3 | **21** | 4 | 3 | 4 | 3 | 4 | 3 | **21** |
| Individual feedback after case of non-implementation is delivered to all staff involved in incident together | 3 | 1 | 3 | 3 | 3 | 3 | **16** | 3 | 2 | 4 | 2 | 3 | 4 | **18** |
| Group feedback sessions are delivered to multi-disciplinary groups (e.g. nurses and doctors together) | 4 | 3 | 4 | 4 | 4 | 4 | **23** | 4 | 3 | 4 | 4 | 4 | 4 | **23** |
| Group feedback sessions include role-play of disagreements over whether “Sepsis Six” should be implemented | 2 | 1 | 1 | 1 | 2 | 2 | **9** | 2 | 1 | 3 | 2 | 3 | 3 | **14** |
| Simulation training is delivered to multi-disciplinary groups (e.g. nurses and doctors together) | 3 | 3 | 4 | 4 | 4 | 4 | **22** | 3 | 2 | 4 | 3 | 4 | 3 | **19** |
| Group feedback sessions include role-play of complicated situations where there is confusion over implementation | 2 | 2 | 2 | 2 | 2 | 2 | **12** | 2 | 1 | 3 | 2 | 3 | 3 | **14** |
| An FAQ document is produced and made available to staff in in break areas and in designated sepsis area (i.e. sepsis trolley) | 4 | 4 | 4 | 4 | 4 | 4 | **24** | 4 | 4 | 4 | 4 | 4 | 4 | **24** |
| Key literature giving evidence for the guidelines are made available to staff in designated sepsis area | 3 | 4 | 4 | 4 | 4 | 4 | **23** | 4 | 3 | 3 | 4 | 4 | 4 | **22** |
| Ward/department monitors attendance at group education, simulation training and feedback sessions | 3 | 3 | 3 | 3 | 4 | 4 | **20** | 3 | 2 | 3 | 4 | 4 | 3 | **19** |
| Protocol checklist includes a tick for handover of patient | 3 | 3 | 3 | 3 | 3 | 3 | **18** | 2 | 2 | 1 | 2 | 2 | 3 | **12** |
| Instruction on how to treat patients who have already received one or more “Sepsis Six” steps is included on protocol document | 3 | 3 | 3 | 3 | 3 | 3 | **18** | 4 | 3 | 2 | 3 | 3 | 3 | **18** |
| Instruction on how to treat patients who have already been treated with “Sepsis Six”, but are showing additional signs is included in protocol document | 3 | 2 | 2 | 3 | 3 | 3 | **16** | 3 | 2 | 2 | 4 | 4 | 3 | **18** |
| “Sepsis Six” education sessions delivered to Hospital at Night Coordinators | 3 | 3 | 3 | 3 | 3 | 3 | **18** | 3 | 3 | 3 | 4 | 4 | 3 | **20** |
| Sepsis bags containing instruments to implement are made available to Hospital at Night Coordinators | 3 | 2 | 2 | 3 | 2 | 3 | **15** | 4 | 4 | 2 | 3 | 3 | 3 | **19** |
| “Sepsis hotline” is created for practical support | 1 | 1 | 1 | 1 | 1 | 1 | **6** | 1 | 1 | 1 | 0 | 1 | 1 | **5** |
| In group education sessions, staff are instructed on how to perform a sepsis crash call at night to request assistance | 2 | 2 | 1 | 2 | 2 | 2 | **11** | 2 | 2 | 1 | 2 | 3 | 2 | **12** |

*Highlighted modifications were included in the final intervention protocol

APPEASE framework reference 23: Michie S, Atkins L, West R. The Behaviour Change Wheel : A Guide to Designing Interventions. London: Silverback; 2014.
