# Supplementary material for: Bridging the gap between pragmatic intervention design and theory: using behavioural science tools to modify an existing quality improvement programme to implement “Sepsis Six”
Source: Implement Sci. 2016 Feb 3;11:14. doi: 10.1186/s13012-016-0376-8 (PMC4739425; doi:10.1186/s13012-016-0376-8)
Supplement: Supplementary file 5 — “Sepsis Six” modified intervention protocol. (DOCX 47 kb) [file 13012_2016_376_MOESM5_ESM.docx]

**“Sepsis Six” Modified Intervention Protocol**

Version 4

**Introduction**

This is the protocol for a modified intervention to increase implementation of the evidence-based “Sepsis Six” clinical care bundle. This intervention is a modified version of an existing intervention which was designed and delivered by a specialist sepsis nursing team at the Royal Free Hospital, London for five years. This modified intervention is informed by theory and data collected about influences on implementation in clinical areas who received the initial intervention ([Steinmo, Fuller, Stone, & Michie, 2015](#_ENREF_1)).

This protocol describes everything that should be delivered to a clinical area implementing the “Sepsis Six”. Most components are delivered by the Patient Safety Facilitator, a member of the sepsis nursing team unless otherwise specified.

Complete and acceptable delivery:

The ideal and complete intervention will consist of the following components, behaviour change techniques and functions. :

| **“Sepsis Six” Intervention Component** | **Functions** |
| --- | --- |
| 1. Partnership agreement | **Enablement** to increase means/reduced barriers to increase capability or opportunity to implement “Sepsis Six” (beyond education and training). |
| 1. Staff introductory education | **Education** to increase knowledge and understanding of sepsis and “Sepsis Six” and **persuasion** to induce positive feelings and stimulate action. |
| 1. Staff training (sepsis simulation) | **Training** to increase capability to implement “Sepsis Six”, impart technical and non-technical skills. |
| 1. Materials and promotional/ educational documents provided | **Environmental restructuring** to change the physical context to increase opportunity to implement “Sepsis Six” and **education** to increase knowledge and understanding of sepsis and “Sepsis Six”. |
| 1. Individual personalised feedback for staff involved in incidents when bundle was not fully implemented | **Enablement** to increase means/reduce barriers to increase capability or opportunity to implement “Sepsis Six” **persuasion** to induce positive feelings and stimulate action. |
| 1. Data audit and group feedback | **Enablement** to increase means/reduce barriers to increase capability or opportunity to implement “Sepsis Six” **persuasion** to induce positive feelings and stimulate action. |
| 1. Hospital at Night Co-ordinator education | **Education** to increase knowledge and understanding of sepsis and “Sepsis Six”. |

1. **How to use this protocol**

The protocol is structured according to the broad component parts of the intervention listed above. Each component section will detail (A) the **aim** of the component; (B) the **delivery details** of the component, including the *mode of delivery*, *who* delivers it, to *whom* and *when/how often*; and (C) the component’s **content**: the text description of what should be delivered and the specific Behaviour Change Techniques (BCTs) applied.

1. **Partnership Agreement**
2. **Aim**

The aim of the *Partnership Agreement* component of the intervention is to engage senior staff members/clinical area leads with “Sepsis Six”, to ensure their commitment to implementation and to agree allocated time for education, training and feedback sessions and access to the clinical area. This agreement is a behavioural contract between The Patient Safety Facilitator and the ward receiving the “Sepsis Six” Plus intervention.

1. **Delivery details**

The *Partnership Agreement* is a written document which the patient safety facilitator and at least two clinical leads on clinical area will work on together and sign. This component is delivered once at start of intervention and amended iteratively as required.

1. **Content**

The precise content of the *Partnership Agreement* may vary between clinical areas according to time, predicted volume of septic patients, staff numbers and resources, but all agreements should include the following:

| ***Partnership Agreement* content** | **BCTs** |
| --- | --- |
| Details of when education and feedback sessions will be delivered, who will attend and who will deliver | Behavioural contract |
| Two sepsis champions (one Doctor and one Nurse) nominated and supported to complete sepsis work | Social support |
| Agreement of commitment to “Sepsis Six” that includes recognition of role model status and that social support will be offered to staff | Identification of self as role-model, social support |
| Agreement that signatories will emphasize expectation of full group engagement with “Sepsis Six” | Commitment, behavioural contract |
| Agreement that signatories will emphasize that challenging others is encouraged | Commitment, behavioural contract |
| Agreement that staff attendance at training and feedback sessions will be recorded | Commitment, Self-monitoring |
| Statement of iterative nature of the Partnership Agreement and recognition that it is a flexible working document and that all parties are involved it its creation and amendments | Commitment |
| Localised/clinical area specific plan for collecting and sharing “Sepsis Six” implementation data including (a) details of who is responsible for data-collection and dissemination to all clinical area staff and the Patient Safety Facilitator, and (b) details of how and where the data will be shared/displayed | Commitment, action planning |
| Agreement that information shared at group feedback sessions will be cascaded down to all staff (those not able to attend feedback sessions) | Commitment, self-monitoring |

1. **Initial education**
2. **Aim**

The aim of the *initial education* component of the intervention is to introduce staff members to the “Sepsis Six”, to increase knowledge and understanding of sepsis and the bundle and begin and to stimulate action towards reaching a clinical area target of 95% implementation for patients that show two or more sepsis triggers and background of infection is either suspected or confirmed. Some questions that staff frequently have about implementation will be answered and some misconceptions about its effects will be clarified.

1. **Delivery details**

*Initial education* sessions are delivered face-to-face by the Patient Safety Facilitator to all Doctors and Nurses in the clinical area. Education sessions are to be delivered until 95% of all staff in the clinical area attended an education session once.

1. **Content**

Where possible, and with the consent of all participants, education sessions should be audio-recorded. Each education session should include the following:

| ***Introductory education* session content** | **BCTs** |
| --- | --- |
| Discussion on severity and susceptibility of sepsis | Information about health consequences |
| Instruction on how & when to implement | Instruction on how to perform a behaviour |
| Story of a young patient who had died from sepsis in the organisation is told | Information about health consequences, salience of consequences |
| Discussion of good implementation on other clinical areas and how this can be reached | Social comparison |
| Evidence for the efficacy of “Sepsis Six” for improving patient outcomes given | Information about health consequences |
| Statement about the achievability of completing the all six steps within one hour | Verbal persuasion about capability |
| Video that features patient experience of having been treated with the bundle and praise for staff who had treated patient | Credible source, salience of consequences, social reward |
| Setting of organisation aim of zero avoidable harm | Goal setting (outcome) |
| Setting of clinical area target of implementation for 95% of patients | Goal setting (behaviour) |
| Statement that staff have legitimate authority to commence “Sepsis Six” using their clinical discretion | Social support (unspecified) |
| Statement that staff are encouraged to seek support from superiors & facilitators regarding implementation issues | Social support (unspecified) |
| Statement that full clinical area involvement is expected | Information about social consequences |
| Statement that challenging others should be un-personal and should be normalised as part of the culture | Generalisation of behaviour |
| Evidence of patient outcomes presented quantitatively in at least two formats | Information about health consequences |
| Instruction on how to perform a sepsis call to Doctor or Hospital At Night Coordinator when help is needed to implement within one hour | Instruction on how to perform a behaviour |
| Statement about importance of using the six steps together as a bundle, not individual components | Information about health consequences |
| Invitation to attend “Sepsis Six” simulation training events | Prompts/cues |
| At least two of following FAQs should be addressed. The Patient Safety Facilitator is to decide which are most relevant to the cohort attending: | Instruction on how to perform a behaviour |
| Fluid volumes including how much is too much for patient and what is meant by STAT | Instruction on how to perform a behaviour |
| Evidence for administering oxygen for all patients | Information about health consequences |
| Broad-spectrum antibiotics including low numbers of *C. Diff* at RFH | Information about health consequences |
| When hour starts for patients already receiving some of the steps of “Sepsis Six”. | Instruction on how to perform a behaviour |
| Evidence for starting “Sepsis Six” as part of wait & see including number needed to harm | Information about health consequences |

1. **Simulation training**
2. **Aim**

The aim of the *simulation training* component of the intervention is to impart technical and non-technical skills to implement “Sepsis Six” more effectively. To normalise and increase confidence and self-efficacy to challenge others’ opinions about whether or not the bundle should be implemented.

1. **Delivery details**

The *simulation sessions* are delivered face-to-face by a member of the simulation faculty to an unspecified proportion of Doctors and Nurses on the clinical area. All doctors and nurses are invited to attend simulation sessions in person (at initial education sessions) and by posters in clinical areas.

**Content**

Each simulation session should include the following:

| ***Simulation training session* content** | **BCTs** |
| --- | --- |
| Observation and practice of a sepsis scenario | Behavioural practice, demonstration of behaviour, modelling |
| Structured de-brief after the sepsis scenario that includes: |  |
| Review of the exercise- what happened | Feedback on behaviour |
| Analysing factors influencing the behaviour and generating strategies that overcome barriers or increase facilitators | Problem solving |

1. ***Documents and materials* provided**
2. **Aim**

The aim of providing documents is to reinforce knowledge and understanding of sepsis and “Sepsis Six” gained during initial education component and to enable implementation by prompting memory of the bundle. The aim of providing materials is to change the physical environment to increase opportunity to implement the bundle.

1. **Delivery details**

Documents refer to written documents (paper and electronic) that are made accessible to all staff on the clinical areas. Materials refer to physical resources or instruments added to the clinical area environment. All staff will have access to all documents and materials. The Patient Safety Facilitator and the nominated sepsis champions are responsible for dissemination and replenishing stocks.

1. **Content**

Documents and materials provided should be as follows:

| ***Documents and materials* content** | **BCTs** |
| --- | --- |
| **The following should be present on the clinical area:** |  |
| A sepsis area containing all materials required to implement bundle that is identifiable and accessible | Adding objects to the environment |
| ‘Sepsis Bags’ are available in PARRT office (for Hospital at Night Coordinator) | Adding objects to the environment |
| Sepsis Six logo (displayed) | Prompts/cues |
| A Sepsis Folder with the following documents inside: |  |
| Antibiotics protocol providing instruction on appropriate antibiotic selection | Instruction on how to perform a behaviour |
| FAQ information sheet | Instruction on how to perform a behaviour |
| Six step checklist for completion of each step (sticker or protocol pathway) | Instruction on how to perform a behaviour, self-monitoring (behaviour) |
| Patient log that lists all patients for whom bundle was commenced | Self-monitoring (behaviour) |

1. ***Individual personalised feedback* for cases of non/partial implementation**
2. **Aim**

The aim of the *individual-level reciprocal feedback* component of the intervention is to target individual staff who have deviated from the standard “Sepsis Six” protocol in their practice. During the sessions, we will aim to explore the reasons for each deviation and share learning about what deviations are acceptable, how the pathway can be developed or refined and to generate strategies to overcome barriers or increase facilitators to implementation going forward.

**B. Delivery details**

*Individual reciprocal feedback* is a two-way conversation that takes place face-to-face between the Patient Safety Facilitator and any staff members who are involved in deviations from standard “Sepsis Six” protocol. This component is therefore delivered ad-hoc with the aim of reaching 80% of staff involved in deviations.

**C. Content**

Where possible, and with the consent of the participant, sessions should be audio-recorded. Each individual-level feedback session should include:

| ***Individual feedback* content** | **BCTs** |
| --- | --- |
| Review of the deviation incident- what happened | Feedback on behaviour |
| Analysis of factors that influenced implementation and if relevant (i.e. if deviation was not acceptable variation) generating strategies that overcome barriers or increase facilitators | Problem solving |

1. ***Data Audit and group feedback***
2. **Aim**

The aim of *ongoing implementation and patient outcome data measurement and group feedback* component of the intervention is to monitor and openly share the clinical area’s progress to clinical area 95% implementation target and increase staff capability and motivation to continue to work toward this goal by addressing barriers, increasing understanding and stimulating positive feelings about the bundle and staff efforts.

**B. Delivery details**

Group feedback sessions are delivered face-to-face by the Patient Safety Facilitator to all nurses and doctors (who will be grouped according to their role). Group feedback sessions should be ongoing over the course of the intervention with the aim of delivering one feedback session per month to available doctors and nurses in the clinical area and reaching a minimum of all clinical leads and sepsis champions.

**C. Content**

Where possible, and with the consent of all participants, group feedback sessions should be audio-recorded. Each group feedback session should include:

| ***Audit and group feedback* session content** | **BCTs** |
| --- | --- |
| Data measurement: |  |
| Implementation data/progress is, e.g. safety cross or run charts, are openly displayed (usually in staff room) | Feedback (behaviour) |
| Trust septic patient outcomes data is measured and openly displayed (usually in staff room) | Feedback (outcome) |
| Group feedback sessions: |  |
| Comparison of current performance with 95% target | Discrepancy between current behaviour and goal |
| Verbal feedback on implementation rates | Feedback (behaviour) |
| Analysis of factors that influenced implementation and if relevant (i.e. if deviation was not acceptable variation) generating strategies that overcome barriers or increase facilitators for better implementation | Problem solving |
| Reporting of patient mortality/morbidity outcomes data | Feedback (outcome) |
| Clinical follow-up for patients who received “Sepsis Six” | Feedback (outcome) |
| Discussion of past targets hit | Focus on past success |
| Praise for improvements made and targets reached | Social reward |

1. ***Hospital at Night Co-ordinator education***
2. **Aim**

The aim of the *Hospital at Night Co-ordinator education* component of the intervention cis to introduce the Hospital at Night Co-ordinators to the “Sepsis Six”, to increase knowledge and understanding of the susceptibility and severity of sepsis in order to stimulate action towards helping staff with “Sepsis Six” implementation on night shifts.

**B. Delivery details**

The *Hospital at Night Co-ordinator education* component is delivered by the Patient Safety Facilitator to all Hospital at Night Co-ordinators. There are twelve in total and we aim to deliver this education once to all in groups or as individuals.

**C. Content**

Each Hospital at Night Co-ordinator education session should include:

| ***Hospital at Night Co-ordinator education* *session* content** | **BCTs** |
| --- | --- |
| Statement about the severity of sepsis | Information about health consequences |
| Statement about the urgency of performing “Sepsis Six” within one hour | Information about health consequences |
| Statement about the importance of finding an on call Dr to attend triggering patients | Information about health consequences |
| Mention of sepsis triggers | Instruction on how to perform a behaviour |
| Mention of available ‘Sepsis Bags’ in the PARRT office | Prompts/cues |

Steinmo, S., Fuller, C., Stone, S. P., & Michie, S. (2015). Characterising an implementation intervention in terms of behaviour change techniques and theory: the "Sepsis Six" clinical care bundle. *Implement Sci, 10*(1). doi: 10.1186/s13012-015-0300-7
